# Maternal Blood, Plasma, and Breast Milk Lead: Lactational Transfer and Contribution to Infant Exposure

**DOI:** 10.1289/ehp.1307187

**Published:** 2013-11-01

**Authors:** Adrienne S. Ettinger, Ananya Roy, Chitra J. Amarasiriwardena, Donald Smith, Nicola Lupoli, Adriana Mercado-García, Hector Lamadrid-Figueroa, Martha Maria Tellez-Rojo, Howard Hu, Mauricio Hernández-Avila

**Affiliations:** 1 Department of Chronic Disease Epidemiology, School of Public Health, Yale University, New Haven, Connecticut, USA; 2 Department of Environmental Health, Harvard School of Public Health, Boston, Massachusetts, USA; 3 Channing Laboratory, Department of Medicine, Brigham and Women’s Hospital, Boston, Massachusetts, USA; 4 Department of Biology and Environmental Toxicology, University of California, Santa Cruz, Santa Cruz, California, USA; 5 Division of Statistics, Center for Evaluation Research and Surveys, National Institute of Public Health, Cuernavaca, Morelos, México; 6 Office of the Director, Dalla Lana School of Public Health, University of Toronto, Toronto, Ontario, Canada; 7 Office of the Director, National Institute of Public Health, Cuernavaca, Morelos, México

## Abstract

Background: Human milk is a potential source of lead exposure. Yet lactational transfer of lead from maternal blood into breast milk and its contribution to infant lead burden remains poorly understood.

Objectives: We explored the dose–response relationships between maternal blood, plasma, and breast milk to better understand lactational transfer of lead from blood and plasma into milk and, ultimately, to the breastfeeding infant.

Methods: We measured lead in 81 maternal blood, plasma, and breast milk samples at 1 month postpartum and in 60 infant blood samples at 3 months of age. Milk-to-plasma (M/P) lead ratios were calculated. Multivariate linear, piecewise, and generalized additive models were used to examine dose–response relationships between blood, plasma, and milk lead levels.

Results: Maternal lead levels (mean ± SD) were as follows: blood: 7.7 ± 4.0 μg/dL; plasma: 0.1 ± 0.1 μg/L; milk: 0.8 ± 0.7 μg/L. The average M/P lead ratio was 7.7 (range, 0.6–39.8) with 97% of the ratios being > 1. The dose–response relationship between plasma lead and M/P ratio was nonlinear (empirical distribution function = 6.5, *p* = 0.0006) with the M/P ratio decreasing by 16.6 and 0.6 per 0.1 μg/L of plasma lead, respectively, below and above 0.1 μg/L plasma lead. Infant blood lead level (3.4 ± 2.2 μg/dL) increased by 1.8 μg/dL per 1 μg/L milk lead (*p* < 0.0001, *R*^2^ = 0.3).

Conclusions: The M/P ratio for lead in humans is substantially higher than previously reported, and transfer of lead from plasma to milk may be higher at lower levels of plasma lead. Breast milk is an important determinant of lead burden among breastfeeding infants.

Citation: Ettinger AS, Roy A, Amarasiriwardena CJ, Smith DR, Lupoli N, Mercado-García A, Lamadrid-Figueroa H, Tellez-Rojo MM, Hu H, Hernández-Avila M. 2014. Maternal blood, plasma, and breast milk lead: lactational transfer and contribution to infant exposure. Environ Health Perspect 122:87–92; http://dx.doi.org/10.1289/ehp.1307187

## Introduction

Human milk has been suggested as a significant potential source of infant lead exposure due to the redistribution of cumulative maternal lead stores associated with bone resorption of pregnancy and lactation (Anderson and Wolff 2000; Silbergeld 1991). Maternal bone lead burden and breastfeeding practices are important predictors of maternal blood lead levels over the course of lactation (Tellez-Rojo et al. 2002); however, plasma lead is the main biologically active compartment from which lead is available to cross cell membranes (Cavalleri et al. 1978; O’Flaherty 1993). The milk-to-plasma (M/P) ratio, used to express the relative efficiency of passive transfer of a chemical from the blood into milk (Larsen et al. 2003), has been reported to be < 1.0 for lead (Lawrence and Lawrence 1998, 2005; Wolff 1983), although human data available to confirm this relationship are limited.

There are some data from rodents on the lactational transfer and uptake of lead in the neonate. Kostial and Momcilovic (1974) showed that the peak transfer of radiolabeled lead in mice from mother to litter occurred during lactation. Keller and Doherty (1980) found that 25% of maternal bone lead burden in mice was transferred to offspring and that most of this activity occurred during lactation. Murine milk was found to concentrate lead at around 25 times the level circulating in plasma.

Because maternal blood is the matrix from which lead is transferred to breast milk and, ultimately, to the breastfeeding infant, the relationship of lead in maternal blood and in breast milk is of key importance. Early studies supported the belief that human milk levels were one-tenth to one-fifth the levels of lead in maternal blood (Abadin et al. 1997). However, Gulson et al. (1998a) and Amarasiriwardena et al. (2013) have discussed in detail the analytical difficulties of quantifying lead in breast milk. More recent studies of breast milk lead have consistently found maternal milk-to-blood lead ratios of ≤ 3% (Counter et al. 2004; Ettinger et al. 2004b; Gulson et al. 1998a; Li et al. 2000). Yet evidence suggests that the plasma-to-blood lead ratio can vary quite widely among and within individuals (Smith et al. 2002), and this may be attributable to underlying differences in toxicokinetics—for example, with respect to δ-aminolevulinic acid dehydratase (ALAD) gene polymorphisms (Montenegro et al. 2006, 2008). Although blood lead levels are highly correlated with plasma lead levels, lead in bone (particularly trabecular bone) exerts an additional independent influence on plasma lead (Hernandez-Avila et al. 1998; Schutz et al. 1996; Smith et al. 1996), and mobilization of lead from bone is amplified during lactation (Gulson et al. 1998b). Thus, the bioavailability of lead for transfer into breast milk may not be well reflected by maternal blood lead levels alone. Better understanding of lead kinetics in the lactating woman and breastfeeding infant are needed for risk assessment and policy development [Centers for Disease Control and Prevention (CDC) 2010; Kosnett et al. 2007; Landrigan et al. 2002] because it is now understood that even low levels of lead are harmful to human health and development (CDC 2012).

To better understand the distribution and transfer of lead during lactation, we measured maternal plasma, blood, and breast milk lead concentrations at 1 month postpartum in a study of lactating women in Mexico City, Mexico. We also explored the relationship between breast milk lead and infant blood lead levels among their breastfeeding infants.

## Materials and Methods

*Study subjects*. Subjects were participants in a longitudinal study of lead biomarkers and reproduction which has been described in detail elsewhere (Lamadrid-Figueroa et al. 2006; Tellez-Rojo et al. 2004). Women were recruited during prenatal visits between May 1997 and July 1999 at one of three clinics of the Mexican Institute of Social Security (IMSS) in Mexico City and followed throughout their pregnancy and for up to 1 year postpartum. All mothers were informed about the study procedures, and those who agreed to participate read and signed a letter of informed consent. The research protocol was approved by the ethics committees of the National Institute of Public Health of Mexico and the participating hospitals, the Harvard School of Public Health, the Brigham and Women’s Hospital, and the University of California, Santa Cruz. A total of 463 pregnant women were recruited. Blood and plasma samples were collected at each trimester and at 1, 4, 7, and 12 months postpartum. Lactating women were also asked to provide breast milk samples at the 1-month postpartum visit. This analysis is limited to 81 subjects for whom there were lead values in breast milk, blood, and plasma at 1 month postpartum. Additionally, among a subset of 60 mother–infant pairs with infant blood lead levels at 3 months of age, we explored the effect of breast milk lead on infant lead burden in this population.

*Plasma lead measurement*. Before venipuncture, each subject’s arm was washed with ultrapure water and disinfected with reagent-grade alcohol. Thirteen milliliters of venous blood was then collected into a polyethylene tube containing 100 USP sodium heparin (H-3393; Sigma Chemical Company, St. Louis, MO, USA) using gravity-assisted procedures to limit potential hemolysis. Samples were processed and shipped frozen to the trace metal facility at the University of California, Santa Cruz, for measurement of plasma lead using ultra-clean methods detailed elsewhere (Hernandez-Avila et al. 1998; Smith et al. 1998). All samples were analyzed using inductively coupled plasma mass spectrometry (ICP-MS; Thermo Finnigan, Bremen, Germany). Potential contamination by lead from hemolyzed red cells was assessed by measuring levels of total plasma iron and free hemoglobin as described by Smith et al. (1998). This method yields a measurement precision of ≤ 0.5% RSD (relative standard deviation) for lead concentrations of > 0.05 ng/mL and an analytical detection limit of 0.01 ng/mL (0.01 μg/L).

*Blood lead measurement*. Maternal and infant venous whole blood was collected into metal-free tubes (Vacutainer, B-D 367734; Becton-Dickinson, Franklin Lakes, NJ, USA) for blood lead analysis. Graphite furnace atomic absorption spectrophotometry (model 3000; PerkinElmer, Norwalk, CT, USA) was used to quantify blood lead according to a technique described by Miller et al. (1987). Measurements were performed at the ABC Hospital Trace Metal Laboratory, which participated in the CDC blood lead proficiency testing program administered by the Wisconsin State Laboratory of Hygiene (Madison, WI). The laboratory standardization program provided external quality control specimens varying from 2 to 88 μg/dL, and our laboratory maintained acceptable performance during the study period. The limit of detection for this method is 0.1 μg/dL (1 μg/L).

*Breast milk lead measurement*. Breast milk samples were collected from lactating women using specific techniques designed to minimize potential for environmental contamination and any potential variability in breast milk lead over time (related to time of day, milking session time, months postpartum). Before manual expression of milk, the breast was washed with deionized water that also was collected and analyzed for lead contamination. Ten milliliters of milk was collected in preleached polypropylene containers. Samples were shipped frozen and stored at –30°C (Fisher IsoTempPlus, New York, NY, USA) until analysis. Breast milk sample preparation was performed at University of Massachusetts Research Institute for Analytical Chemistry (Amherst, MA), and instrumental analysis was performed at the Trace Metals Laboratory of Harvard School of Public Health. Digestion was performed using nitric acid in high temperature high pressure asher (HPA) (Anton Paar USA, Ashland, VA, USA), and lead content in the samples was analyzed by isotope dilution–inductively coupled plasma mass spectrometry (ID-ICPMS) (Sciex Elan 6100-DRC; Perkin Elmer, Norwalk, CT, USA) by methods previously described in detail (Amarasiriwardena et al. 2013; Ettinger et al. 2004b). The precision for these measurements ranged from 0.3 to 7.8% RSD, and the analytic detection limit by this method is 0.01 ng/mL (0.01 μg/L).

*Statistical analysis*. Univariate distributions were examined for all variables. Outliers were identified as values > 3 SDs from the mean: Subjects with blood lead levels > 30 μg/dL (*n* = 1) and plasma lead levels > 1 μg/L (*n* = 2) were excluded. Milk and plasma lead concentrations are expressed in parts per billion (ppb), whereas blood lead concentrations, typically expressed in micrograms per deciliter, were converted to micrograms per liter as necessary to calculate transfer indices (plasma–blood lead ratio, breast milk–blood lead ratio). The M/P ratio was calculated by dividing breast milk lead concentration (micrograms per liter) by plasma lead concentration (micrograms per liter).

Bivariate analyses were carried out to explore the relationships between the different biomarkers of lead exposure. Spearman tests of correlation were used and correlation coefficients (ρ) with *p*-values reported. Nonparametric smoothing [locally weighted scatterplot smoothing (LOWESS), bandwidth 0.75] was used to explore the shape of the associations between the different lead biomarkers.

Multivariable linear regression models were fitted using ordinary least-squares methods to estimate associations between the blood or plasma and breast milk lead levels. Given the limited sample size available for analysis, extensive covariate adjustment was not feasible. We chose to investigate covariates (maternal age, dietary calcium intake, and systolic blood pressure) based on *a priori* knowledge of biological significance and review of the scientific literature. Covariates were retained in the models if the main effect estimate of plasma or blood lead on breast milk lead changed by > 10% or if it increased the model coefficient of determination (*R*^2^) by > 10%. To explore potential nonlinear associations, we also examined the relationships between the variables using nonparametric regression with generalized additive models (GAMs). The GAM models allowed us to examine possible nonlinear effects using a smooth functional term corresponding to plasma lead in the multivariate model. The smoothing term was fitted by using penalized regression splines with degree of smoothness selected by cross-validation technique, allowing us to determine the functional relationship between the different matrices in a flexible, data-adaptive way instead of being restricted to a linear relationship. Where the GAMs indicated a nonlinear relationship, we explored the effects using piecewise linear models to describe the trend.

We explored the contribution of breast milk lead to infant blood lead levels among a subset of mother–infant pairs using multivariate regression accounting for breastfeeding duration (months of exclusive breastfeeding) over the preceding 3 months of the infant’s life. Stratified and interaction models were also fitted to determine whether breastfeeding status (yes/no, exclusively breastfed in the preceding month) altered this relationship. Sensitivity analyses of the breast milk–infant blood lead association were carried out to assess the additional contribution of lead from umbilical cord blood (where available) among 36 mother–infant pairs, accounting for infant birth weight (as a proxy for the amount of milk consumed) and breastfeeding status. All statistical analyses were performed using SAS® for Windows, version 9.3 (SAS Institute Inc., Cary, NC, USA) and R Programming Language (version 2.12.2; R Development Core Team, Vienna, Austria).

## Results

Maternal lead levels at 1 month postpartum were as follows: blood: 7.7 ± 4.0 (range, 1.7–28.7) μg/dL; plasma: 0.1 ± 0.1 (range, 0.03–0.5) μg/L; and breast milk: 0.8 ± 0.7 (range, 0.04–3.2) μg/L. The plasma-to-blood ratio ranged from 0.06 to 0.42, and the average M/P lead ratio was 7.7 (range, 0.6–39.8) with 97% of the ratios being > 1. Infant blood lead at 3 months of age was 3.4 ± 2.2 (range, 0.5–14.5) μg/dL (Table 1). Significant correlation between the different lead biomarkers was observed (Table 2).

**Table 1:** Summary of mother–infant lead levels in different matrices and transfer indices, Mexico City, 1997–1999.

Lead matrix or transfer index	*n*	Mean ± SD	Minimum	Maximum
Maternal (1 month postpartum)
Blood lead (μg/dL)	81	7.7 ± 4.0	1.7	28.7
Plasma lead (μg/L)	81	0.1 ± 0.1	0.03	0.5
Breast milk lead (μg/L)	81	0.8 ± 0.7	0.04	3.2
Plasma–blood lead ratio	81	0.2 ± 0.1	0.06	0.42
Breast milk–blood lead ratio	81	1.2 ± 0.9	0.1	4.2
Breast milk–plasma lead ratio	81	7.7 ± 7.1	0.6	39.8
Infant (3 months of age)
Blood lead (μg/dL	60	3.4 ± 2.2	0.5	14.5

**Table 2:** Correlation matrix^*a*^ for mother–infant lead biomarkers, Mexico City, 1997–1999.

Biomarker of lead exposure	Breast milk^*b*^	Blood^*b*^	Plasma^*b*^	Blood^*c*^
Maternal
Breast milk	1.00	0.44*	0.31**	0.34**
Blood		1.00	0.76*	0.65*
Plasma			1.00	0.44**
Infant blood				1.00
^***a***^Spearman correlation coefficients (ρ); prob > |*r*| under H_0_: ρ = 0. ^***b***^Maternal: sample collected 1 month postpartum, *n* = 81. ^***c***^Infant blood: sample collected at 3 months of age, *n* = 60. *p < 0.0001. **p < 0.01.				

**Figure 1:**
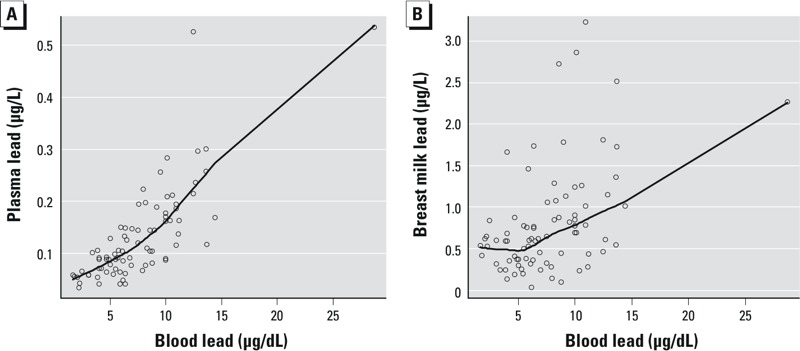
Unadjusted associations between maternal blood lead and maternal plasma (*A*) and breast milk lead (*B*) levels. The solid line represents a smoothed curve through the set of individual data points (open circles) from a robust locally weighted regression (bandwidth = 0.7).

**Figure 2:**
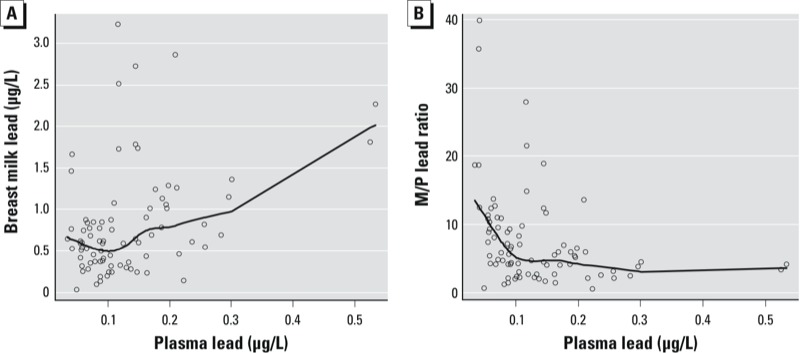
Unadjusted associations between maternal plasma lead and (*A*) maternal breast milk lead and (*B*) breast M/P ratio. The solid line represents a smoothed curve through the set of individual data points (open circles) from a robust locally weighted regression (bandwidth = 0.7).

The multivariate linear relationships, adjusted for maternal age, dietary calcium intake and systolic blood pressure, between maternal blood lead, plasma lead, and breast milk lead are reported in Table 3. Figure 1A suggests a curvilinear association between blood and plasma lead after excluding outliers; however, the association best fit a log-linear model. A 1-μg/dL increase in blood lead was associated with an increase of 0.11 (95% CI: 0.09, 0.13) natural log (ln) (micrograms per liter) plasma lead and explained 57% of the variation in plasma lead (*p* < 0.0001); however, the multivariate adjusted GAM model indicated that the association was linear [empirical distribution function (EDF) = 1] (data not shown). As Figures 1B and 2A indicate, blood and plasma lead levels have nonlinear associations with breast milk lead. Therefore, to describe the association between plasma and breast milk lead, we additionally fit a piecewise linear model with a knot value of 0.1 μg/L. The change in breast milk lead per 0.1-μg/L increase of plasma lead was –2.0 (95% CI: –7.4, 3.5) μg/L when plasma lead levels were < 0.1 μg/L, and 1.6 (95% CI: 0.1, 3.2) μg/L when plasma lead levels were > 0.1 μg/L. The difference in slope was marginally significant in the piecewise linear model (*p* = 0.05). Figure 2B shows that the plasma lead to M/P ratio relationship (indicative of relative transfer of lead) varied over the observed range of plasma lead levels.

**Table 3:** Multivariate^*a*^ linear associations between lead levels in maternal plasma and maternal blood, and between lead levels in breast milk and lead in maternal blood or plasma.

Outcome	Exposure	β (95% CI)	SE	*R*^2^	*p*-Value^*b*^
Plasma^*c*^	Blood	0.1 (0.09, 0.1)	0.01	0.6	< 0.0001
Breast milk^*d*^	Blood	0.1 (0.04, 0.1)	0.02	0.2	< 0.0001
Breast milk^*e*^	Plasma	2.3 (1.0, 3.6)	0.7	0.2	0.001
Breast milk^*f*^	Plasma: ≤ 0.1 μg/L	–2.0 (–7.4, 3.5)	2.7	0.2	0.05
	Plasma: > 0.1 μg/L	1.6 (–0.1, 3.2)	0.9
^***a***^All models adjusted for maternal age, dietary calcium intake, and systolic blood pressure. ^***b***^*R*^2^ *p*-value for models of maternal blood or plasma as continuous variables, or *p*-value for the difference in coefficients (slopes) > 0.1 and ≤ 0.1 μg/dL maternal plasma lead. ^***c***^Linear regression of maternal plasma lead [ln(μg/L)] and maternal blood lead (μg/dL). ­^***d***^Linear regression of breast milk lead (μg/L) and maternal blood lead (μg/dL). ^***e***^Linear regression of breast milk lead (μg/L) and maternal plasma lead (μg/dL). ^***f***^Linear regression of breast milk lead (μg/L) and maternal plasma lead as a piecewise linear variable with knot value at 0.1 μg/L.					

**Figure 3:**
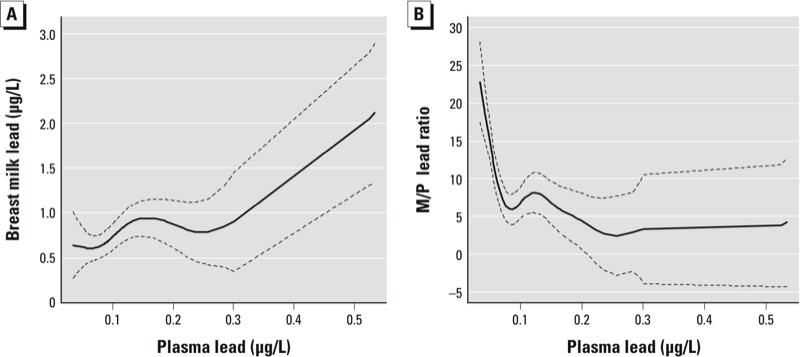
Nonlinear dose–response relationship between plasma lead and breast milk lead (EDF = 4.2) (*A*) and M/P ratio (EDF = 6.5) (*B*), from GAMs using penalized splines and adjusted for maternal age, dietary calcium intake, and systolic blood pressure. The M/P ratio decreased by 16.6 (95% CI: –24.6, –8.7) and 0.6 (95% CI: –2.5, 1.2) per 0.1‑μg/L plasma lead below and above 0.1 μg/L plasma lead, respectively, in a piecewise linear multivariate model. Solid lines represents effect estimates and dashed lines represent 95% CIs.

The adjusted plasma–breast milk lead relationship was observed to be nonlinear with considerable variability over the range of plasma lead levels (Figure 3A; EDF = 4.2). The M/P ratio was also nonlinear (EDF = 6.5) and changed over the plasma lead range (Figure 3B). Below and above 0.1 μg/L the M/P ratio decreased, respectively, by 16.6 (95% CI: –24.6, –8.7) and 0.6 (95% CI: –2.5, 1.2) per 0.1 μg/L plasma lead. The difference in slope was statistically significant in the piecewise linear model (*p* = 0.002).

**Figure 4:**
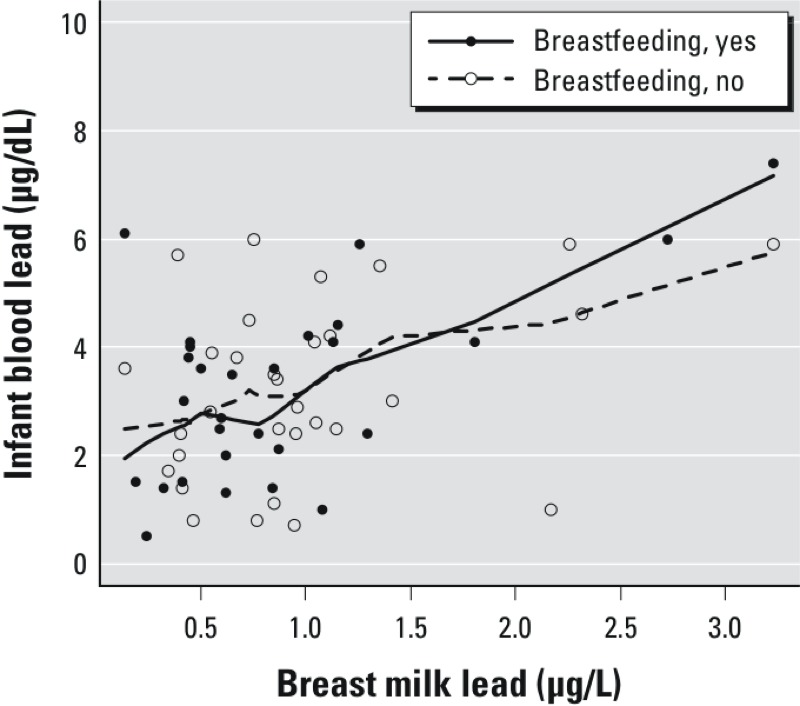
Unadjusted association between breast milk lead and infant blood lead stratified by breastfeeding status for infants exclusively (yes) and not exclusively breastfeeding (no) in preceding month. Lines represent smoothed curve through the set of individual data points from a robust locally weighted regression (bandwidth = 0.7).

Infant blood lead was significantly associated with maternal breast milk levels (Table 4). Breast milk lead explained 30% of the variation in infant blood lead levels. After accounting for number of months of breastfeeding, infant blood lead at 3 months of age increased by 1.8 (95% CI: 1.1, 2.6) μg/dL per μg/L breast milk lead. Further, infant blood lead increased by 2.2 (95% CI: 1.1, 3.3) μg/dL among infants exclusively breastfed in the preceding month, compared with 1.1 (95% CI: 0.01, 2.2) μg/dL among those not breastfed in the preceding month (*p*-value for interaction = 0.06), indicating effect modification by breastfeeding status (Figure 4).

**Table 4:** Relationship between breast milk lead (μg/L, 1 month postpartum) and infant blood lead (μg/dL, 3 months of age).

Predictor	Estimate (95% CI)	*p*-Value	Partial *R*^2^
Unadjusted (*n* = 60)
Breast milk	1.8 (1.1, 2.6)	< 0.0001	0.3
Adjusted (*n* = 60)
Breast milk	1.8 (1.1, 2.6)	< 0.0001	0.3
Breastfeeding (months)	0.3 (–0.6, 0.8)	0.8	0.0
Stratified model^*a*^
Breastfed in preceding month (*n *= 29)			
Breast milk	2.2 (1.1, 3.3)	0.0001	0.4
Not breastfed in preceding month (*n *= 31)
Breast milk	1.1 (0.01, 2.2)	0.04	0.1
^***a***^*p* = 0.06 for interaction between breast milk lead and breastfeeding status.			

Sensitivity analyses indicate that this relationship remained even after accounting for umbilical cord blood lead and birth weight; the increase in infant blood lead per unit change in breast milk lead was higher by 2.3 μg/dL among children exclusively breastfed compared with those not exclusively breastfed in the previous month (*p*-value for interaction = 0.06) (data not shown). Among infants exclusively breastfed, breast milk and umbilical cord blood explained 37% and 33% of variation in infant blood lead, respectively, compared with 10% and 50% of the variation among infants not exclusively breastfed in the preceding month.

## Discussion

The results of this study suggest that the maternal toxicokinetics of lead between the compartments of blood, plasma (the bioavailable fraction), and breast milk (the exposure medium for breastfeeding infant) are complex. The relationship between maternal blood and plasma in our population had a curvilinear association best described by a log-linear function, suggesting variability in partitioning of lead between the erythrocyte component of blood and plasma over the range of lead exposure. This is very similar to that observed among 63 nonpregnant (Smith et al. 2002) and 237 pregnant women in Mexico (Lamadrid-Figueroa et al. 2006). Plasma lead increases as a fraction of blood lead. The plasma–blood lead ratio in our population varied from about 0.1 to 0.4, and the dose–response across the range of blood lead levels (data not shown) was also similar to the hockey stick–shaped association seen in the study by Smith et al. (2002). Therefore, the amount of lead available for transfer from plasma into breast milk varied over the range of blood lead, so blood lead may not be the best matrix in which to study the kinetics of lead transfer into breast milk and, ultimately, as a source of exposure to the breastfeeding infant.

Our results suggest that the transfer of lead from plasma to breast milk was also variable across the range of plasma lead. The M/P ratios reported here for lead are higher than previously estimated in humans as ≤ 1.0, and may actually be more similar to results reported by earlier animal studies where lead concentrated in breast milk. In fact, the M/P ratio for lead was found to be nonlinear, with the highest M/P ratios at the lowest levels of plasma lead, which dropped off steeply until around 0.1 μg/L plasma lead and then tapered off. This could be explained by passive transfer of lead into breast milk driven by high affinity compartment in milk, thus allowing binding at low concentrations and drawing lead into milk. However, this high affinity compartment would have a low overall capacity, leading to rapid saturation at around 0.1 μg/L plasma lead and the consequent leveling off of the M/P ratio as passive transfer resumes (driven only by the increase in plasma lead levels).

A possible candidate for this high-affinity, low-capacity compartment in milk could be the protein casein, which is involved in calcium transport into milk. Several studies indicate that milk casein binds lead with high affinity in rats, mice, cows, and humans [reviewed by Anastacio Ada et al. (2004)]. However, the milk casein concentration and amount of binding to casein in milk is much higher in animals than in humans (Hallen and Oskarsson 1995; Lönnerdal 2013; Rosen et al. 2009), which would explain the ease with which binding becomes saturated in human milk (Oskarsson et al. 1995).

Current recommendations for cessation of breastfeeding at maternal blood lead levels > 40 μg/dL are based on an M/P ratio assumed to be < 1 for lead (CDC 2010; Kosnett et al. 2007; Lawrence and Lawrence 1998, 2005). We found that, in most cases (97%), the M/P lead ratio was > 1, indicating that the milk lead concentration is higher than the maternal plasma concentration. Although the M/P ratios up to 40 observed in our study might seem to be disturbingly high, in fact they occur only at such low plasma lead concentrations that the associated milk lead concentration is still relatively low. For example, the extreme of the observed M/P ratios in our study, 39.8, corresponds to a milk lead concentration of 1.7 μg/L at a plasma lead concentration of 0.04 μg/L. Even at a higher and more commonly observed plasma lead concentration of 0.1 μg/L, an M/P ratio of 40 would correspond to a milk lead concentration of 4 μg/L.

At high blood lead concentrations (> 40 μg/dL), the relationship between the concentration of lead in serum and its concentration in blood becomes nonlinear (Manton and Cook 1984; Manton et al. 2001). The blood lead concentration at which deviation from linearity in the plasma-blood lead relationship is clearly apparent is both population- and model-dependent but, in general, it appears that blood lead concentrations > 40 μg/dL are associated with significant nonlinearity in humans (Hernandez-Avila et al. 1998; O’Flaherty 1993; Schutz et al. 1996). Thus, it can be posited that when maternal blood lead concentrations exceed about 40 μg/dL, the milk-to-blood lead ratio may be greater than it is at lower maternal blood lead concentrations.

The proposed nonlinearity in the milk-to-blood lead relationship has been observed in lactating mice and rats with blood lead concentrations > 3 μg/dL (Hallén et al. 1996) as well as in cows at blood lead concentrations exceeding 20–30 μg/dL (Oskarsson et al. 1992). Hallén and Oskarsson (1993) observed a linear relationship between milk lead and maternal plasma lead in rats and mice throughout the entire concentration range of their studies. Oskarsson et al. (1995) have also noted a rapid rise in milk lead above blood lead levels > 40 μg/dL in human samples from a Mexico City study (Namihira et al. 1993), in which the mean blood lead was 46 μg/dL, suggesting a curvilinear relationship between blood lead and milk lead. The variance explained in the linear models of breast milk lead are approximately the same for blood and plasma lead in our study; however, we found the blood–breast milk lead association to be linear while the plasma–breast milk lead was significantly nonlinear. Therefore, we fit piecewise linear models, with a knot at the point of inflection of 0.1 μg/L, to describe the plasma–breast milk lead relationship, which may not fully be capturing the variability of the nonlinear relationship as shown in Figures 2A and 3A.

The potential magnitude and range of increases in the milk-to-blood lead ratio at maternal blood lead concentrations > 40 μg/dL are not well quantified in humans and are outside the range of blood lead levels in our current study. However, although the milk-to-blood lead ratio may increase as maternal blood lead concentrations exceed 40 μg/dL, the milk-to-plasma lead ratio should not change significantly at higher maternal blood lead concentrations. Nonetheless, the possibility that milk lead increases disproportionately to blood lead in women with high blood lead levels should be considered when discussing breastfeeding with women whose blood lead levels exceed 40 μg/dL.

Our findings here do not suggest that measurement of maternal plasma or breast milk lead are likely to become clinically useful environmental health measures because these methods require technically challenging ultra-clean specimen collection and analysis protocols. However, when properly collected and analyzed, these biological markers of exposure are useful tools in research efforts to better understand lead kinetics in the lactating woman and breastfeeding infant. We used different laboratories and analytic procedures for quantification of lead in the various biological matrices to employ the best available methods for the key matrices of interest (plasma and breast milk). Blood lead is a surrogate, measured with error, for the toxicologically available lead fraction, and larger effect size estimates observed in studies using plasma lead suggest many previous studies using blood lead may have had effect estimates downwardly biased by measurement error.

Given the unique nutritional characteristics of human milk, breastfeeding is understood to be the optimal mode of nutrient delivery to full-term infants. The benefits of breastfeeding are so compelling that very few situations definitively contraindicate breastfeeding (American Academy of Pediatrics 2012). However, wide ranges of breast milk lead levels have been documented in population studies of women (Abadin et al. 1997; Koyashiki et al. 2010), and the human data available to estimate the risk this poses to the breastfeeding infant are limited (Mushak 1999).

Our results suggest that breast milk lead is a significant and important source of infant lead exposure accounting for approximately 30% of variation in infant blood lead levels. This was higher among infants who were exclusively breastfed (37%) than among those not exclusively breastfed (10%), suggesting that breast milk is an important contributor to infant lead burden on top of any concurrent environmental and *in utero* exposures. Although a limitation of the present study is that we did not have concurrent infant blood lead levels available at 1 month postpartum, we made the assumption that the ranking of lead levels within the population remained stable over the first 3 months of infant life, and we also accounted for breastfeeding practices in our models.

Previously we have reported that even among a population of women with relatively high cumulative lifetime exposures to lead, levels of lead in breast milk were low, influenced both by current lead exposure and by redistribution of bone lead accumulated from past environmental exposures (Ettinger et al. 2004b). Although these breast milk lead levels were relatively low, they clearly had a strong influence on infant blood lead over and above the influence of maternal blood lead. Breast milk lead accounted for 12% of the variance, and was significantly correlated (*r* = 0.32, *p* < 0.0001) with infant blood lead levels at 1 month of age (Ettinger et al. 2004a). In that study, we estimated that a difference of approximately 2 μg/L in breast milk lead was associated with a 0.82-μg/dL increase in blood lead for breastfeeding infants at 1 month of age (Ettinger et al. 2004a). In the current study, a 1-μg/L increase in breast milk lead increased infant blood lead by 1.8 μg/dL at 3 months of age (*p*-value < 0.0001). This was higher among infants exclusively breastfed in the previous month (2.2 μg/dL) compared with breastfeeding infants who were not exclusively breastfed in the preceding month (1.1 μg/dL) (*p*-value for interaction = 0.06).

In another study of breast milk and infant blood lead levels, milk lead accounted for 10% of the variance in 6-month blood lead, and there was a linear dose–response relationship between breast milk and infant blood lead at 6 months of age (*r* = 0.42, *p* = 0.0003) (Rabinowitz et al. 1985). Despite the fact that human milk composition varies among individuals (Ballard and Morrow 2013), the high percentage of lead in the milk whey fraction suggests that most lead in human milk is bioavailable to the breastfeeding infant (Anastacio Ada et al. 2004). To the extent that lead can be found in infant formula, the relative bioavailability of such lead may be less than that of lead in breast milk. For example, it has been well documented that iron is more readily absorbed from breast milk than from infant formula (Lönnerdal 1985). Rabinowitz et al. (1985) found breast milk to be the strongest correlate of 6-month blood lead levels, whereas formula lead correlated poorly with infant blood lead levels. However, Gulson et al. (1998a) showed that the contribution of formula to infant blood lead varied from 24% to 68% in exclusively formula-fed infants. Further, a study of breastfeeding duration and infant blood lead reported that longer breastfeeding was associated with higher infant lead concentrations in three countries, in three different decades, in settings with differing breastfeeding patterns, environmental lead sources, and infant lead levels (Lozoff et al. 2009). Nonetheless, lead in water used to reconstitute powdered infant formula (Baum and Shannon 1997) and other dietary intakes are also potential sources of lead exposure to infants beyond the contribution from lead in breast milk.

## Conclusions

In this study we measured lead in several maternal and infant biomarkers simultaneously, including blood, plasma, and breast milk, using state-of-the-art, ultra-clean methods for specimen collection and laboratory analysis. We found that the M/P ratio for lead in humans is substantially higher than previously reported as being ≤ 1.0, and the transfer of lead from plasma to breast milk may be higher at lower levels of plasma lead. Breast milk represents an additional important source of lead exposure to breastfeeding infants over and above the contribution from *in utero* exposure. This has implications for policy decisions regarding counseling the lead-exposed woman on breastfeeding, because current recommendations appear to be based on limited empirical evidence.

## References

[r1] Abadin HG, Hibbs BF, Pohl HR (1997). Breast-feeding exposure of infants to cadmium, lead, and mercury: a public health viewpoint.. Toxicol Ind Health.

[r2] Amarasiriwardena CJ Jayawardene I Lupoli N Barnes RM Hernandez-Avila M Hu H 2013 Comparison of digestion procedures and methods for quantification of trace lead in breast milk by isotope dilution inductively coupled plasma mass spectrometry. Anal Methods 5 1676 1681; 10.1039/C3AY26321E PMC4010228 24808927

[r3] American Academy of Pediatrics. 2012 Breastfeeding and the use of human milk. Pediatrics 129 3 e827 e841; 10.1542/peds.2011-3552 22371471

[r4] Anastacio Ada S, da Silveira CL, Miekeley N, Donangelo CM (2004). Distribution of lead in human milk fractions: relationship with essential minerals and maternal blood lead.. Biol Trace Elem Res.

[r5] Anderson HA, Wolff MS (2000). Environmental contaminants in human milk.. J Expo Anal Environ Epidemiol.

[r6] Ballard O, Morrow AL (2013). Human milk composition: nutrients and bioactive factors.. Pediatr Clin North Am.

[r7] Baum CR, Shannon MW (1997). The lead concentration of reconstituted infant formula.. J Toxicol Clin Toxicol.

[r8] Cavalleri A, Minoia C, Pozzoli L, Polatti F, Bolis PF (1978). Lead in red blood cells and in plasma of pregnant women and their offspring.. Environ Res.

[r9] CDC (Centers for Disease Control and Prevention). (2010). Guidelines for the Identification and Management of Lead Exposure in Pregnant and Lactating Women.. http://www.cdc.gov/nceh/lead/publications/LeadandPregnancy2010.pdf.

[r10] CDC (Centers for Disease Control and Prevention). (2012). Low-Level Lead Exposure Harms Children: A Renewed Call for Primary Prevention.. http://www.cdc.gov/nceh/lead/acclpp/final_document_010412.pdf.

[r11] Counter SA, Buchanan LH, Ortega F (2004). Current pediatric and maternal lead levels in blood and breast milk in Andean inhabitants of a lead-glazing enclave.. J Occup Environ Med.

[r12] Ettinger AS Tellez-Rojo MM Amarasiriwardena C Bellinger D Peterson K Schwartz J 2004a Effect of breast milk lead on infant blood lead levels at 1 month of age. Environ Health Perspect 112 1381 1385; 10.1289/ehp.6616 15471729 PMC1247564

[r13] Ettinger AS Tellez-Rojo MM Amarasiriwardena C Gonzalez-Cossio T Peterson KE Aro A 2004b Levels of lead in breast milk and their relation to maternal blood and bone lead levels at one month postpartum. Environ Health Perspect 112 926 931; 10.1289/ehp.6615 15175184 PMC1242024

[r14] Gulson BL, Jameson CW, Mahaffey KR, Mizon KJ, Patison N, Law AJ (1998a). Relationships of lead in breast milk to lead in blood, urine, and diet of the infant and mother.. Environ Health Perspect.

[r15] Gulson BL, Mahaffey KR, Jameson CW, Mizon KJ, Korsch MJ, Cameron MA (1998b). Mobilization of lead from the skeleton during the postnatal period is larger than during pregnancy.. J Lab Clin Med.

[r16] Hallén IP, Norrgren L, Oskarsson A (1996). Distribution of lead in lactating mice and suckling offspring with special emphasis on the mammary gland.. Arch Toxicol.

[r17] Hallén IP, Oskarsson A (1993). Dose dependent transfer of ^203^lead to milk and tissue uptake in suckling offspring studied in rats and mice.. Pharmacol Toxicol.

[r18] Hallén IP, Oskarsson A (1995). Bioavailability of lead from various milk diets studied in a suckling rat model.. Biometals.

[r19] Hernandez-Avila M, Smith D, Meneses F, Sanin LH, Hu H (1998). The influence of bone and blood lead on plasma lead levels in environmentally exposed adults.. Environ Health Perspect.

[r20] Keller CA, Doherty RA (1980). Lead and calcium distributions in blood, plasma and milk of the lactating mouse.. J Lab Clin Med.

[r21] Kosnett MJ Wedeen RP Rothenberg SJ Hipkins KL Materna BL Schwartz BS 2007 Recommendations for medical management of adult lead exposure. Environ Health Perspect 115 463 471; 10.1289/ehp.9784 17431500 PMC1849937

[r22] Kostial K, Momcilovic B (1974). Transport of lead 203 and calcium 47 from mother to offspring.. Arch Environ Health.

[r23] Koyashiki GA, Paoliello MM, Tchounwou PB (2010). Lead levels in human milk and children’s health risk: a systematic review.. Rev Environ Health.

[r24] Lamadrid-Figueroa H, Tellez-Rojo MM, Hernandez-Cadena L, Mercado-Garcia A, Smith D, Solano-Gonzalez M (2006). Biological markers of fetal lead exposure at each stage of pregnancy.. J Toxicol Environ Health A.

[r25] Landrigan PJ, Sonawane B, Mattison D, McCally M, Garg A (2002). Chemical contaminants in breast milk and their impacts on children’s health: an overview.. Environ Health Perspect.

[r26] Larsen LA, Ito S, Koren G (2003). Prediction of milk/plasma concentration ratio of drugs.. Ann Pharmacother.

[r27] Lawrence R, Lawrence R. (1998). Breastfeeding: A Guide for the Medical Profession, 5th ed. St.

[r28] Lawrence R, Lawrence R. (2005). Breastfeeding: A Guide for the Medical Profession, 6th ed. St.

[r29] Li PJ, Sheng YZ, Wang QY, Gu LY, Wang YL (2000). Transfer of lead via placenta and breast milk in human.. Biomed Environ Sci.

[r30] Lönnerdal B (1985). Biochemistry and physiological function of human milk proteins.. Am J Clin Nutr.

[r31] Lönnerdal B (2013). Bioactive proteins in breast milk.. J Pediatr Child Health.

[r32] Lozoff B Jimenez E Wolf AW, Angelilli mL, Zatakia J, Jacobson SW, et al. 2009 Higher infant blood lead levels with longer duration of breastfeeding. J Pediatrics 155 5 663 667 10.1016/j.jpeds.2009.04.032 PMC3118670 19595371

[r33] Manton WI, Cook JD (1984). High accuracy (stable isotope dilution) measurements of lead in serum and cerebrospinal fluid.. Br J Ind Med.

[r34] Manton WI, Rothenberg SJ, Manalo M (2001). The lead content of blood serum.. Environ Res.

[r35] Miller DT, Paschal DC, Gunter EW, Stroud PE, D’Angelo J (1987). Determination of lead in blood using electrothermal atomisation atomic absorption spectrometry with a L’vov platform and matrix modifier.. Analyst.

[r36] Montenegro MF, Barbosa F, Sandrim VC, Gerlach RF, Tanus-Santos JE (2006). A polymorphism in the delta-aminolevulinic acid dehydratase gene modifies plasma/whole blood lead ratio.. Arch Toxicol.

[r37] Montenegro MF, Barbosa F, Tanus-Santos JE (2008). Assessment of how pregnancy modifies plasma lead and plasma/whole blood lead ratio in ALAD 1-1 genotype women.. Basic Clin Pharmacol Toxicol.

[r38] Mushak P 1999 Response: Keeping abreast of new science [Letter] Environ Health Perspect 107 A59 A61 10348626 10.1289/ehp.107-1566334 PMC1566334

[r39] Namihira D, Saldivar L, Pustilnik N, Carreon GJ, Salinas ME (1993). Lead in human blood and milk from nursing women living near a smelter in Mexico City.. J Toxicol Environ Health.

[r40] O’Flaherty EJ (1993). Physiologically based models for bone-seeking elements. IV. Kinetics of lead disposition in humans.. Toxicol Appl Pharmacol.

[r41] Oskarsson A, Jorhem L, Sundberg J, Nilsson NG, Albanus L (1992). Lead poisoning in cattle—transfer of lead to milk.. Sci Total Environ.

[r42] Oskarsson A, Palminger Hallen I, Sundberg J (1995). Exposure to toxic elements via breast milk.. Analyst.

[r43] Rabinowitz M, Leviton A, Needleman H (1985). Lead in milk and infant blood: a dose-response model.. Arch Environ Health.

[r44] Rosen JM Woo SLC Comstock JP 2009 Regulation of casein messenger RNA during the development of the rat mammary gland. J Mammary Gland Biol Neoplasia 14: 343 351 19653075 10.1007/s10911-009-9143-7

[r45] Schutz A, Bergdahl IA, Ekholm A, Skerfving S (1996). Measurement by ICP-MS of lead in plasma and whole blood of lead workers and controls.. Occup Environ Med.

[r46] Silbergeld EK (1991). Lead in bone: implications for toxicology during pregnancy and lactation.. Environ Health Perspect.

[r47] Smith D, Hernandez-Avila M, Tellez-Rojo MM, Mercado A, Hu H (2002). The relationship between lead in plasma and whole blood in women.. Environ Health Perspect.

[r48] Smith DR, Ilustre RP, Osterloh JD (1998). Methodological considerations for the accurate determination of lead in human plasma and serum.. Am J Ind Med.

[r49] Smith DR, Osterloh JD, Flegal AR (1996). Use of endogenous, stable lead isotopes to determine release of lead from the skeleton.. Environ Health Perspect.

[r50] Tellez-Rojo MM, Hernandez-Avila M, Gonzalez-Cossio T, Romieu I, Aro A, Palazuelos E (2002). Impact of breastfeeding on the mobilization of lead from bone.. Am J Epidemiol.

[r51] Tellez-Rojo MM, Hernandez-Avila M, Lamadrid-Figueroa H, Smith D, Hernandez-Cadena L, Mercado A (2004). Impact of bone lead and bone resorption on plasma and whole blood lead levels during pregnancy.. Am J Epidemiol.

[r52] Wolff MS (1983). Occupationally derived chemicals in breast milk.. Am J Ind Med.

